# Midwifery-led researches for evidence-based practice: Clinical midwives engagement in research in Ethiopia, 2021

**DOI:** 10.1371/journal.pone.0268697

**Published:** 2022-06-03

**Authors:** Keflie Yohannes Gebresilassie, Adhanom Gebreegziabher Baraki, Belayneh Ayanaw Kassie, Sintayehu Daba Wami

**Affiliations:** 1 Midwifery Directorate, University of Gondar, Gondar, Ethiopia; 2 Department of Epidemiology and Biostatistics, University of Gondar, Gondar, Ethiopia; 3 Midwifery Directorate, School of Midwifery, University of Gondar, Gondar, Ethiopia; 4 Department of Environmental and Occupational Health and Safety, University of Gondar, Gondar, Ethiopia; Gulu University, UGANDA

## Abstract

**Introduction:**

Health workers involvement in research had an impact on studies and whole system. They influence the clinical practice and help to implement evidences. Although International Confederation of Midwives (ICM) put research as one of the midwifery competencies and professional development activity, clinical midwives are poorly involved in research. Therefore, this study is aimed to assess clinical midwives engagement in research and bridge the gap through applicable strategies.

**Method:**

Institution-based cross-sectional study was conducted among clinical midwives working at public health facilities of Central and North Gondar Zone, Ethiopia from September to October, 2020 G.C. A structured and pre-tested self-administered questionnaire was used to collect data and entered into Epi-info version 7. Descriptive statistics was used to describe study population. Bi-variable and multi-variable logistic regression analysis was performed using STATA Version 14 and significance level declared at 95% confidence interval, p-value ≤ 0.05 and respective odds ratios.

**Result:**

Out of 335 clinical midwives, 314 were participated making the response rate 93.7%. Among the midwives, one hundred seventy two (54.8%) (95% CI: 49.08%, 60.37) have good skill on conducting a research. Clinical midwives with mothers with formal education [AOR: 1.90, 95% CI: (1.03, 3.51), currently work on referral hospitals [AOR: 2.33, 95% CI: (1.19, 4.53)] and having good level of knowledge on research [AOR: 2.19, 95% CI: (1.25, 3.82)] have significant association with having good research skill. Forty eight (15.2%) (95% CI: 11.5%, 19.7%) ever participated in research during their clinical practice. Clinical midwives who have good knowledge on research [AOR: 0.31, 95% CI: (0.14, 0.70)] are about 0.3 times less likely to participate on research than who have poor knowledge [AOR: 0.31, 95% CI: (0.14, 0.70)].

**Conclusion and recommendation:**

Although more than half have good research skill, only a small proportion of midwives were involved in research. Capacity building activities are crucial to strengthen midwives skill on research and ensure their involvement.

## Introduction

Health workers involvement in research had an impact on studies and whole system. They influence the clinical practice and help to implement evidences [1]. The new approach named clinical academics had health care and academic roles, thus they combine practice with research [2]. Despite these recommendations [3], most college and universities didn’t have clinical academic [4] and they are not appropriately utilizing their potentials.

Health workers involved in research activities have various reasons that includes individual interest, as part of the curriculum, to improve service quality through shred of evidences, prior experience and/or exposure, professional development and financial benefits [1]. Nevertheless, International Confederation of Midwives (ICM) has put continuous professional development including research activities as one of midwifery competencies [5].

Clinical midwives perceived research as other professions role, especially the academic [6]. They had to aware of and involve in research to improve the clinical care [7] and overall quality of midwifery services as they can identify health problems for research from their experience.

Although research capacity building for clinical midwives is recommended [8], most involve as data collector and not more than that. Individuals were capacitated with training, support, workshops and using technologies. In low and middle-income countries projects, partnership and network had built health research capacity. However the lack of empirical research has become a challenge to see their effectiveness [9].

Once ability to influence practice with research, difficulties to work with the academics [1], and communication skills could affect their motivation [10] and confidence [11] to conduct research were individual barriers for conducting a research. Organizational leadership and management and research recognitions [1] also had an effect on research capacity. Resources for research such as dedicated time [12, 13], research expertise [14], access to research findings [15] and opportunities [1]; availability of funding [12, 14, 16] and investment on research activity [15, 17] could limit once research capacity and ability to conduct research. Other studies added that building research partnerships [10], having research culture [16], professional development opportunities and inadequate salaries [14, 15] as cause to poorly involve in research. At Supra-organizational level, health research policies and governance [10] had an influence on participation and involvement in research.

Despite the observed gaps and limiting factors, scientific studies are lacking to study clinical midwives engagement in research and contributing factors. Thus, this study was done to bridge the gaps, which will help to set appropriate strategies and interventions to conduct midwifery-led researches. The study will be a baseline for conducting further studies and results will have an input for School of Midwifery at University of Gondar to improve the curriculum and built midwifery student’s research capacity at undergraduate level.

## Methodology

### Study design, setting, study population and sampling

Institution-based cross-sectional study was conducted among clinical midwives working at public health facilities of Central and North Gondar Zone, Ethiopia, from September to October 2020 G.C. The study area covers two of the four zones of Amhara region (Central, west, north and south Gondar Zones), in which around 6,335,757 estimated populations are living. There are a total of 23 public hospitals and 222 health center. In North and Central Gondar Zone, around 350 trained registered clinical midwives are working in these institutions. All Midwives working in clinical setting of Central and North Gondar Zone were considered as the source and study population. All registered midwives working in the study area were included, whereas those who are working in administrative and academic area, midwives who are sick and unable to respond were excluded from the study.

### Data collection and quality control

Before actual data collection, discussion was done on prevention measures of the current pandemic, Corona-Virus (Covid-19) and basic protective materials (Sanitizer, face mask and glove) were given for data collectors and supervisors. A structured pre-tested self-administered questionnaire was used to collect the data. The tool was developed by referring different literatures [18], first prepared in English and translated back into Amharic, the local language. The tool was checked for consistency statistically using Cronbach’s alpha. Training was given for five data collectors and supervisor on the objective of the study and confidentiality for two days. Pretest was done on 5% of sample size among midwives working other than the study area and necessary correction done. The collected data was assessed for completeness and accuracy on daily basis. The tool has socio-demographic and academic characteristics; questions for assessing research skill and participation. Clinical Midwives are a registered midwives working in the clinical setting/area. A participant who answers more than 50% of the skill assessment questions will be considered as having good skill on research. Similarly, a participant will be considered as practicing (conducting) research if s/he has ever involved in part of a research other than one conducted as a partial fulfillment of his or her midwifery study.

### Data management and analysis procedure

Data was entered into Epi-info version 7 and exported to STATA version 14 for further analysis. Descriptive analysis like frequencies, percentages, means and standard deviations computed for all variables. Model fitness was tested with Hosmer and Lemeshow goodness of fit and both bi-variable and multivariate logistic regression models were carried out to estimate the association. Variables with a p -value of less than 0.2 in the bi-variable analysis were entered into the multivariable logistic regression analysis. Both Crude Odds Ratio (COR) and Adjusted Odds Ratio (AOR) with their corresponding 95% confidence intervals were estimated. Finally, variables with a P-value of less than 0.05 in multivariable logistic regression model were considered as significantly associated with knowledge and attitude towards research.

## Result

### 1. Socio demographic and academic characteristics

Out of 335 clinical midwives 314 were participated making the response rate 93.7%. Age of the midwives range from 18 to 50 years, with median age of 27 years old. More three fifth (66.9%) of the midwives age was between 25 to 29 years. Among all midwives, more than half (52.9%) were male, while two hundred seventy four (87.3%) were Urban dwellers. More than three fifth (63.1%) of the midwives’ were Bachelor degree holders, while majority (73.6%) were graduated from governmental colleges. Nearly there fifth of the midwives (58.6%) study with regular educational program (Table 1).

**Table 1:** Socio-demographic and academic characteristics of clinical midwives working at public health facilities of central Gondar zone, 2020.

Variable	Number (#)	Percentage (%)
**Age (in years)**		
Less than or equal to 24	35	11.2
25–29	210	66.9
30 and above	69	21.9
**Sex**		
Male	166	52.9
Female	148	47.1
**Religion**		
Orthodox Christian	293	93.3
Muslim	19	6.1
Protestant	2	0.6
**Residence**		
Urban	274	87.3
Rural	40	12.7
**Mother Educational Status**		
No formal Education	237	75.5
Have Formal education	77	24.5
**Father Educational Status**		
No formal Education	220	70.1
Have Formal education	94	29.9
**Highest educational qualification**		
Diploma (level IV)	98	31.2
Degree	198	63.1
Masters and above	18	5.7
**Type of school/facility you are graduated from**		
Governmental	231	73.6
Private	83	26.4
**Program of study you accomplished**		
Regular	184	58.6
Extension	130	41.4
**Taking Prior Research Course**		
Yes	216	68.8
No	98	31.2
**Level of health facility that you are currently working**		
Specialized/Referral Hospital	93	29.6
General/Primary Hospital	71	22.2
Health Center	150	47.8
**Current working unit** *(sum exceed the total sample and 100% due multiple responses)*		
Labor and delivery Room	220	70.1
Family planning Room	100	31.8
Comprehensive Abortion Care Room	53	16.9
Antenatal Care Room	138	43.9
Others Specify**	20	6.4
**Years of experience as a clinical midwife (in Year)**		
<2 year	43	13.7
2–4	114	36.3
>4	157	50
**Average Monthly Income (in Ethiopian Birr)**		
<4000	41	13.1
4000 and above	184	58.6
Not willing to mention	89	28.3

Others*—dead

Others**—Gyn ward, Postnatal care, Youth Friendly Service, Immunization

### 2. Clinical midwives research skill and practice

#### 2.1 Skill of clinical midwives to conduct a research

Among the midwives, one hundred seventy two have good skill on conducting a research making the magnitude 54.8% (95% CI: 49.08%, 60.37).

Among the midwives, nearly half (48.4%) reported as having high skill on identifying research problems, while 132 (42%) have high skill on conducting literature review. More than two fifth (42.7%) and one hundred twenty three (39.2%) of the midwives reported as having poor skill on data management and data analysis using software respectively. Clinical midwives reported as they have high skill on applying for research funding (35%) and to give advice for less experienced researchers (28.7%). (Table 2).

**Table 2:** Clinical midwives skill to conduct a research at public health facilities of north and central Gondar Zones, Northwest Ethiopia, 2020.

Variable	Poor skill	Moderate Skill	High Skill
Identify research Problems	93 (29.6%)	69 (22%)	152 (48.4%)
Conduct literature Review	90 (28.7%)	92 (29.3%)	132 (42%)
Tool development and data collection	93 (29.6%)	65 (20.7%)	156 (49.7%)
Data management using software	134 (42.7%)	60 (19.1%)	120 (38.2%)
Conduct Data analysis	123 (39.2%)	78 (24.8%)	113 (36%)
Interpret analyzed data	110 (35%)	83 (26.4%)	121 (38.5%)
Write discussion and conclusion	109 (34.7%)	77 (24.5%)	128 (40.8%)
Put references using software	95 (30.3%)	83 (26.4%)	136 (43.3%)
Write manuscript for publication	133 (42.4%)	92 (29.3%)	89 (28.3%)
Present research findings in conferences	108 (34.4%)	81 (25.8%)	125 (39.8%)
Give advice for less experienced researchers	125 (39.8%)	99 (31.5%)	90 (28.7%)
Applying for research funding	102 (32.5%)	102 (32.5%)	110 (35%)

*2*.*1*.*1*. *Factors associated with clinical midwives skill on research*. To identify factors, bi-variable and multi-variable logistic regression analysis was carried out for seven explanatory variables. In multi-variable analysis; Mother educational status of having formal education; currently working on referral health facilities; having good level of knowledge on research and taking prior research course have a positive significant association with skill on research (Table 3).

**Table 3:** Bi-variable and multi-variable logistic regression analysis output of factors associated with clinical midwives knowledge research at central and north Gondar public health facilities, Northwest Ethiopia, 2020.

Variable	Level of research skill		Crude Odds Ratio [95% CI]	Adjusted Odds Ratio [95% CI]	P-value
	Good	Poor			
**Age**					
Less than equal to 24	17 (5.4%)	18 (5.7%)	1	1	
25 to 29	106(33.8%)	104(33.1%)	1.08 [0.53, 2.21]	0.95 [0.43, 2.08] 1.52	
30 and above	49 (15.6%)	20 (6.4%)	2.59 [1.12, 6.02]	[0.60, 3.85]	
**Mother Edu. Status**					
No formal education	116 (36.9%)	121 (38.5%)	1	1	
Have formal Education	56 (17.8%)	21 (6.7%)	2.78 [1.58, 4.88]	**1.90 [1.03, 3.51]**	**0.04**
**Facility type graduated from**					
Governmental	146 (46.5%)	85 (27.1%)	3.77 [2.21, 6.43]	1.56 [0.82, 2.97]	
Private	26 (8.3%)	57 (18.2%)	1	1	
**Level of facility currently working**					
Referral Hospitals	70 (22.3%)	23 (7.3%)	4.44 [2.50, 7.87]	**2.33 [1.19, 4.53]**	
General/Primary Hospital	41 (13.1%)	30 (9.6%)	1.99 [1.13, 3.54]	1.77 [0.93, 3.38]	**0.01**
Health Center	61 (19.4%)	89 (28.3%)	1	1	
**Taking prior research course**					
Yes	144 (45.9%)	72 (22.9%)	5.00 [2.97, 8.42]	1.95 [0.99, 3.82]	0.05
No	28 (8.9%)	70 (22.3%)	1	1	
**Level of Knowledge on Research**					
Good	109 (34.7%)	45 (14.3%)	3.73 [2.33, 5.97]	**2.19 [1.25, 3.82]**	
Poor	63 (20.1%)	97 (30.9%)	1	1	**0.006**
**Ever participate in research**					
Yes	35 (11.1%)	13 (4.1%)	2.54 [1.28, 5.01]	1.26 [0.59, 2.71]	
No	137 (43.6%)	129 (41.1%)	1	1	

Clinical midwives who take prior research course were about 1.9 times more likely to have good research skill than their counterparts. [AOR: 1.95, 95% CI: (1.00, 3.82)].

Clinical midwives who have mothers with formal education are about 1.9 times more likely to have good research skill. [AOR: 1.90, 95% CI: (1.03, 3.51)].

Clinical midwives who currently work on specialized/referral hospitals were about 2.3 times more likely to have good research skill than their counterparts. [AOR: 2.33, 95% CI: (1.19, 4.53)].

Clinical midwives who have good level of knowledge on research are about 2.2 times more likely to have good research skill than their counterparts. [AOR: 2.19, 95% CI: (1.25, 3.82)].

#### 2.2. Clinical midwives involvement in conducting research

**Fig 1:**
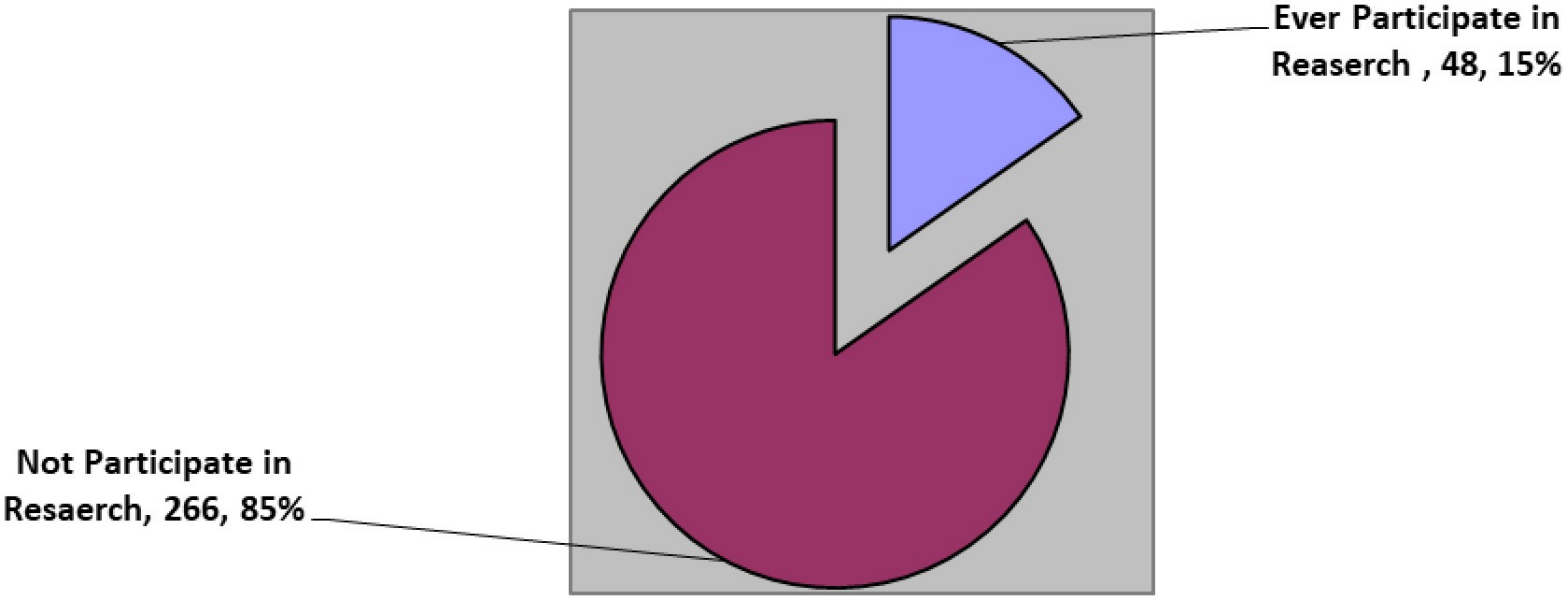
Clinical midwives practice on research at public health facilities of central and North Gondar Zone, Northwest Ethiopia, 2020.

Among all the midwives, forty eight (15.2%) (95% CI: 11.5%, 19.7%) ever participated in research during their clinical practice. (Fig 1).

More than half (52.1%) have involved in one research activities, while thirty (9.6%) have a responsibility of data collection in the research they involved. Nearly three fifth (72.9%) believe that the research they involved in contributed to the policy and/or the clinical practice in any way. Among the midwives, twenty three (7.3%) ever present at conferences and 13 (4.1%) ever publish research findings. (Table 4).

**Table 4:** Clinical midwives practice on conducing research at public health facilities of central and North Gondar Zone, Northwest Ethiopia, 2020.

Variable	Number	Percentage
**Ever participate in research during your clinical practice? (n = 314)**		
Yes	48	15.2
No	266	84.8
**In how many research projects have you been involved?** (n = 48)		
One	25	52.1
Two and above	23	47.9
**What was your responsibility in the research activity you involved?**		
Coordination of a research project	18	5.7
Selection of research problems	27	8.6
Review of the literature	18	5.7
Recruitment of participants	17	5.4
Data collection	31	9.9
Data management and analysis	11	3.5
Writing research report / manuscript preparation	16	5.1
**Who was the leader of research you have participated? (n = 48)**		
Midwife	34	70.8
Physicians/Doctors	17	35.4
Nurse and Other public health professionals	27	32.6
**Do you believe that the research you involved in contributed to the policy and/or the practice in any way?**		
Yes	35	72.9
No	13	27.1
**Have you ever present a research findings at conferences? (n = 314)**		
Yes	23	7.3
No	291	92.7
**Have you ever publish a research findings? (n = 314)**		
Yes	13	4.1
No	301	95.8
**If yes, how many (n = 13)**		
One	8	61.5
Two and above	5	38.5

*2*.*2*.*1*. *Factors associated with clinical midwives practice on a research*. To identify factors, bi-variable and multi-variable logistic regression analysis was carried out for five explanatory variables that have association with outcome variable. In multi-variable analysis; knowledge level on research course have a negative significant association with participation on research. (Table 5).

**Table 5:** Bi-variable and multi-variable logistic regression analysis output of factors associated with clinical midwives practice on research at central and north Gondar public health facilities, Northwest Ethiopia, 2020.

Variable	Participate on Research		Crude Odds Ratio [95% CI]	Adjusted Odds Ratio [95% CI]	P-value
	Yes	No			
**Sex**					
Male	33 (10.5%)	133 (42.4%)	0.46 [0.24, 0.88]	0.55 [0.27, 1.10]	
Female	15 (4.8%)	133 (42.4%)	1	1	
**Facility type graduated from**					
Governmental	44 (14%)	187(59.6%)	0.22 [0.08, 0.62]	0.57 [0.18, 1.80]	
Private	4 (1.3%)	79 (25.2%)	1	1	
**Taking prior research course**					
Yes	45 (14.3%)	171 (54.5%)	0.12 [0.34, 0.40]	0.30 [0.80, 1.11]	
No	3 (1.0%)	95 (30.3%)	1	1	
**Knowledge level on Research**					
Good	39 (12.4%)	115 (36.6%)	0.18 [0.08, 0.38]	**0.31 [0.14, 0.70]**	**0.005**
Poor	9 (2.9%)	151 (48.1%)	1	1	
**Level of research skill**					
Good	35 (11.1%)	137 (43.6%)	0.39 [0.20, 0.78]	0.72 [0.34, 1.50]	
Poor	13 (4.1%)	129 (41.1%)	1	1	

Clinical midwives who have good knowledge on research are about 0.3 times less likely to participate on research than who have poor knowledge. [AOR: 0.31, 95% CI: (0.14, 0.70)].

## Discussion

The ICM strongly recommends involvement of midwives in research to provide high quality midwifery services [19]. This study was conducted to assess clinical midwife’s engagement on research and associated factors in Northwest Ethiopia. A total of three hundred fourteen midwives working at public health facilities were participated and majority (63.1%) were registered midwives with Bachelor degree holders.

Only nearly above half (50.6%) of the midwives say that their facility has continuous professional development program for staffs including midwives and this indicate that there is a limited opportunity to upgrade oneself. Unless there is no adequate and continual support to midwives, quality of midwifery services provided for the women could be affected [20]. A study in Tanzania was also evident that lack of evidence-based practices supported with research could result to poor service provision for patients (30% to 40%) and to have poor health outcomes [21]. A recent studies review highlighted that midwifery and nurses research publication are increased and suggested to have capacity building activities for strengthening the observed result [22]. Although it is not found significant, level of income is associated with quality of midwives performance on provision of care as evidenced by a study conducted in Gaza [23]. Professional benefits such as good salary might have an effect on midwives motivation and retention.

A significant proportion (52.9%) of midwives also responded that their health facilities doesn’t conduct research activities relevant to clinical practice. This might be due to that majority (64.5%) of midwives work on Primary Health Care units (Health Centers and Primary Hospitals). In Ethiopian health care system, facilities are not expected to conduct research activities unless they have teaching role, in addition to patient care service [24]. Midwives also reported that in addition to poor support from their facility (63.1%) and other professionals (60.2%), there are no opportunities to participate in research conferences (52.9%). As a result midwives poorly utilized research findings in their clinical service [25]. Moreover lack of dedicated time and poor implementation of research findings further deteriorate the application of research in the clinical practice [26].

In our study higher odds of good research skill was noted among midwives with formal maternal education (1.9 times) and it has an effect on academic performance [27].

Midwives who work on specialized/referral hospitals were found to have higher good research skill (2.3 times) than who work in primary health care facilities (health centers). This finding is supported with recent study conducted in North Gondar [25] and might be reasoned with that in referral health facilities there might be different opportunities to learn about research and related activities as they are teaching hospitals. Moreover these facilities are more likely to utilize research findings in their day too day clinical practice [25].

Having good research knowledge was associated with having good skill on research (2.2 times). Both research knowledge and skill are crucial to conduct a research as they are interrelated competencies.

Our study found that a small proportion of midwives (15.2%) ever participated in research during their clinical practice, in which 9.6% as data collector. This indicate that there is limited opportunities for midwives to be involved in research activities. Although a higher proportion (36.4%) of Australian nurses were reported as they involved in research, there is still a deficiency in health professional’s engagement in research activities [28]. In Latin America and the Caribbean, a review of studies also found that there is gap on midwifery-led researches, where most (95.8%) studies were nurses-led [29]. In our study, although more than three fifth (70.8%) of the midwives reported as they participated in a midwives-led researches, their capacity could be improved if they have the opportunity to work collaboratively with other disciplines such as public health experts, epidemiologists and physicians. Nearly three fifth (72.9%) believe that the research they involved in contributed to the policy and/or the clinical practice in any way. This is indicate that midwives have a positive understanding on the research activities they involved in. As they know the practical setting, they can identify and suggest on the real problem that will benefit the woman and her child [7].

Although midwives have good knowledge on research, they were less likely (0.3 times) to ever participate on research than who have poor knowledge. This indicate that there is limited opportunities for midwives with adequate research knowledge. This might be due to that a significant proportion of midwives (47.8%) work on health centers and opportunities are scarce.

## Conclusion

The study find that research capacity of clinical midwives is not adequate. Only small proportion (15.2%) of midwives participated in research and having good knowledge on research was associated with it. Similarly research skill was associated with mothers with formal education, currently working in specialized /referral health facilities, and having good knowledge on research.

### Recommendation

Ethiopian Ministry of Health better to capacitate health facilities to conduct local researches, particularly primary and general hospitals. It is also better to give priority and support health professionals working in the clinical setting to conduct research and related activities. With the existing continuous professional development programs, Regional Health Bureau better to expand opportunities for clinical midwives working in the region.

Ethiopian Midwifery Association (EMwA), University of Gondar and School of Midwifery better to contribute a lot to support clinical midwives with capacity building activities on research such as training, create opportunities and arrange conferences so that they can be involved and conduct researches in their clinical practice. Strengthening the integration of the school and hospital midwifery coordinator is also crucial to work collaboratively and share experience on research and related activities. Moreover it is good to provide dedicated time for clinical service providers including midwives to participate in research and related activities.

## Supporting information

S1 Dataset (DTA)
